# Yellow Fever

**Published:** 1888-09

**Authors:** 


### YELLOW FEVER.

The occurrence of yellow fever in the city of Jacksonville, Fla., has created an amount of excitement and interest seemingly out of proportion to the extent of the epidemic and the rapidity of the spread of the disease. A whole city has gone into exile, and a score or more of other cities have barred their gates against the fleeing multitudes before the number of deaths has reached a half dozen. Other cities in the same State have been visited by the same disease without exciting more than passing remark, but as soon as it is declared to be epidemic in Jacksonville, the whole country is thrown into a state of agitation. The reason for this apparent anomaly is found in the fact that Jacksonville is the largest city that has been visited by the disease in several years; that it is a favorite winter resort for visitors from all parts of the United States, and that it is more closely connected with other parts of the country by railroad communication than any other city in Florida.

On account of the present widespread interest in the subject, we publish in this number of THE JOURNAL an article by Dr. J. P Wall, of Tampa, embodying the results of a study of over one hundred cases which have come under the observation of the author. While this article presents little that is absolutely new in regard to the disease, it brings out more clearly than has been done by previous authors certain points which bear an important relation to the diagnosis, prognosis and treatment of yellow fever. It shows quite conclusively that, in the present state of our knowledge, our therapeutical resources are of little avail in the direction of combatting and curing the disease, but at the same time it presents a more hopeful view of the prognosis than has heretofore been generally entertained—a view which is corroborated by the course of the present epidemic in Jacksonville.

The action of the authorities of Atlanta in throwing open her gates and inviting the refugees from her fever-stricken sister city is one which has in some quarters excited a considerable amount of adverse criticism. It has been claimed that such action is based upon sentiment alone—a factor which is obviously out of place in the discussion of a question which involves the safety of seventy thousand people. We believe, however, that such criticism is unjust, and that the action of our city fathers is based upon far more solid grounds than those referred to. An examination of the subject will show that there are valid reasons why Atlanta has no cause to fear an epidemic of yellow fever. There are conditions existing in our city which place it upon a different plane from any city or town which has heretofore been visited by the disease. These conditions are found in its elevation above the sea and its topographical situation. The history of the disease in the past has shown that no city of the altitude of Atlanta has ever been ravaged by yellow fever, and that those cities which have been so afflicted have been situated either upon the seaboard or upon some water course. We do not recall a single exception to these rules. It is true that future experience may prove that what we have taken for rules may be mere coincidence. But the same might be said of any conclusions based upon the experience of the past.

If these conclusions be accepted, our sympathy for our suffering neighbors is timely and well directed, and the action based upon it is honorable alike to the heads and hearts of those under whose control the matter has been placed. The measures adopted are such as are dictated equally by humanity and by common sense, and, therefore, should meet with the approval and support of all good citizens. We trust, theretore, that in the future, as in the past, our gates may remain wide open, and that Atlanta may remain a haven of refuge for the weary, suffering and afflicted from whatsoever quarter they may come.

### ITEMS.

AT the annual banquet of the Atlanta Society of Medicine, Dr. J. S. Todd, in replying to the toast, "The Physicians," said among other things:

But a short while since a great statesman breathed his last in New York. While living this man was the avowed enemy, the unrelenting, domineering foe of my people and her institutions; yet we, because of his honesty and ability, were proud to point to him as a fellow-countryman. I hope earthly glory is not all the reward in store for Conklin g, but I do know that his patient physician, the Christian scientist, Agnew, who passed over the river but a short while after, now wears a crown which will grow brighter and brighter even after time merges into eternity. Yet Agnew is accidentally known ephemerally to the masses because he was Conkling's physician.

In the great final reckoning, I had rather be A. W. Calhoun than Julius Cæsar. The one will be welcomed, garlanded, crowned and glorified by the thousands to whom while living he gratuitously gave or restored sight, hearing and smell; the other, execrated by the thousands whom he slew, will hear through all the ages, instead of glad shouts, the widow's wail and the orphan's cry.

I had rather present as a passport for entrance to the pearly gates the receipt which Caventou and Pelletier used to rob cinchona of its quinine than the blood-stained swords of Charlamagne or Napoleon. I had rather be, in the ages to come, the spirit of Jenner or Lister than that of either Cromwell or Wellington. The discovery of Jenner has been freighted with more good to the human family than the finding of a new world by the greatest of navigators, Columbus. By vaccination tens of thousands are rescued yearly from the most painful and loathsome of all deaths. The finding of the two Americas entailed upon the aborigines slavery or extermination.

The writings of Hippocrates and Galen have done more to make life tolerable than the combined books of Homer, Virgil, Confucius, Plato and all the other ancient authors, if you will—with, among the moderns, Darwin, Huxley, Tindall, Mill and Buckle thrown in.

If of W. F. Westmoreland and L. A. Dugas it is not written that they "loved the Lord," then I am sure the recording angel will plead before a merciful tribunal the fact that their works bear mute testimony to the truth of his chronicle, "they loved their fellow-men."

Dr. F. W. McRae has been appointed Lecturer on Diseases of Children in the Atlanta Medical College.

The medical fraternity of Johnson county, Mo., adopted the following resolution: "After January 1, 1888, no account will be allowed to run over six months from date of first visit without satisfactory settlement. All accounts are due when services are rendered. Parties who are in the habit of running bills from one year to another without paying must continue to employ their former physician until he is payed in full, or pay cash for every visit in advance to the new one, charity cases excepted." The State Society of Arkansas has adopted a similar rule, and the law sustains them. If such rules were general all over the United States, would it not be a mercy to the people by compelling them to stand by their physician long enough to give the patient a thorough course of treatment, as well as teach people to pay their honest debts?—*Medical Times.*

